# *Gardenia jasminoides* Attenuates Allergic Rhinitis-Induced Inflammation by Inhibiting Periostin Production

**DOI:** 10.3390/ph14100986

**Published:** 2021-09-28

**Authors:** Bo-Jeong Pyun, Joo Young Lee, Yu Jin Kim, Kon-Young Ji, Dong Ho Jung, Ki-Sun Park, Kyuhyung Jo, Susanna Choi, Myung-A Jung, Yun Hee Kim, Taesoo Kim

**Affiliations:** 1 KM Convergence Research Division, Korea Institute of Oriental Medicine, 1672 Yuseong-daero, Yuseong-gu, Daejeon 34054, Korea; bjpyun@kiom.re.kr (B.-J.P.); jy0130@kiom.re.kr (J.Y.L.); jinjin0228@kiom.re.kr (Y.J.K.); jky8387@kiom.re.kr (K.-Y.J.); jdh9636@kiom.re.kr (D.H.J.); jopd7414@kiom.re.kr (K.J.); susannachoi@kiom.re.kr (S.C.); jma3430@kiom.re.kr (M.-A.J.); 2 KM Science Research Division, Korea Institute of Oriental Medicine, 1672 Yuseong-daero, Yuseong-gu, Daejeon 34054, Korea; kisunpark@kiom.re.kr

**Keywords:** allergic rhinitis, inflammation, *Gardenia jasminoides*, reactive oxygen species, cytokines, T-helper type 2 cell

## Abstract

Allergic rhinitis (AR) is a chronic inflammatory condition affecting the nasal mucosa of the upper airways. Herein, we investigated the effects of extracts from *Gardenia jasminoides* (GJ), a traditional herbal medicine with anti-inflammatory properties, on AR-associated inflammatory responses that cause epithelial damage. We investigated the inhibitory effects of water- and ethanol-extracted GJ (GJW and GJE, respectively) in an ovalbumin-induced AR mouse model and in splenocytes, differentiated Th2 cells, and primary human nasal epithelial cells (HNEpCs). Administering GJW and GJE to ovalbumin-induced AR mice improved clinical symptoms including behavior (sneezing and rubbing), serum cytokine levels, immune cell counts, and histopathological marker levels. Treatment with GJW and GJE reduced the secretion of Th2 cytokines in Th2 cells isolated and differentiated from the splenocytes of these mice. To investigate the underlying molecular mechanisms of AR, we treated IL-4/IL-13-stimulated HNEpCs with GJW and GJE; we found that these extracts significantly reduced the production of mitochondrial reactive oxygen species via the uncoupling protein-2 and periostin, a biomarker of the Th2 inflammatory response. Our results suggest that GJ extracts may potentially serve as therapeutic agents to improve the symptoms of AR by regulating the Th2 inflammatory response of the nasal epithelium.

## 1. Introduction

Allergic rhinitis (AR), a chronic inflammatory disorder of the upper airways, is frequently associated with asthma and has a major impact on global health [1]. AR is a hypersensitivity response resulting from immunoglobulin E (IgE)-mediated inflammation upon the contact of specific allergens with the nasal mucosa, characterized by symptoms such as nasal itching, rubbing, sneezing, lacrimation, and rhinorrhea [2,3,4,5]. Common triggers of AR include environmental allergens such as dust mites, pollen, grass, and pet fur [6].

Allergen exposure induces the recruitment, accumulation, and activation of various immune cells (such as eosinophils, mast cells, basophils, and neutrophils) within the nasal mucosa, resulting in the activation of epithelial cells and fibroblasts [7,8,9]. Moreover, the recruitment and activation of eosinophils concomitant with the allergic inflammatory response is regulated by T-helper type 2 (Th2) cells and cytokines, especially interleukin (IL)-4, IL-5, and IL-13 [6,7]. These cellular events trigger the generation of antigen-specific IgE from B cells [6], which in turn activates mast cells and basophils, leading to the release of histamine and other inflammatory mediators such as leukotrienes and prostaglandins [10,11,12]. Eventually, hypersensitivity and inflammation of the nasal mucosa ensue, culminating in AR symptoms. As the interface between the host and environment as well as the point of initiation of allergic responses, the respiratory airway epithelium has an important role in host defense and regulating immune responses given that it is the first line of defense against inhaled irritants, pathogens, and allergens [13,14]. As such, controlling the immune responses in the airway epithelium could be an effective strategy for managing inflammation caused by rhinitis, asthma, and other airway diseases [15,16].

Previous studies have suggested that periostin, stimulated by IL-4 or IL-13, is secreted by several types of airway cells [17,18,19,20]. Originally named osteoblast-specific factor 2, periostin is an extracellular matrix protein and, along with eosinophils, is a biomarker of Th2 inflammation in allergic diseases [20,21]. Moreover, periostin is highly expressed in the sub-epithelial sites of patients with chronic inflammatory eosinophilic airway diseases; periostin plays an essential role in the pathogenesis of allergic diseases [17,18,19,20] and has been reported to regulate fibrosis, collagen deposition, and tissue remodeling in allergic airway inflammation [22,23,24].

*Gardenia jasminoides Ellis* (GJ) is an evergreen shrub belonging to the Rubiaceae family that is also known in Chinese as “Zhi Zi”. It grows in several Asian countries including China, Korea, Taiwan, Japan, and India [25], and is also commonly used as an organic yellow dye and traditional herbal medicine [25]. Previous studies have shown that GJ fruits have antipyretic, diuretic, and detoxifying effects, and are widely used as antiphlogistic, analgesic, and anti-inflammatory agents. Moreover, they have antiangiogenic, antidiabetic, anticancer, antioxidant, and anti-depressant properties while also improving sleep quality [25,26,27]. The major constituents of GJ include iridoid glycosides and their aglycones (genin) as well as bioactive compounds such as gardenoside, geniposide, genipin, and crocin [28,29]. Crocin is a chemical compound of water-soluble carotenoids known as gardenia yellow and has potent antioxidant properties. Hence, GJ containing crocin has been reported to inhibit the expression of various inflammatory factors in animal models of allergic inflammatory diseases such as asthma and atopic dermatitis [30,31] while improving allergy symptoms in afflicted patients [32].

However, the mechanisms underlying the allergen-induced nasal epithelial inflammatory responses of the upper airways have not been fully elucidated. Therefore, in the present study, we examined the effects of GJ extracts on pathophysiological changes in the nasal mucosa of mice with ovalbumin-induced AR. We also investigated their effects on the nasal mucosal epithelia using IL-4- and IL-13-stimulated primary human nasal epithelial cells (HNEpCs) and on mitochondrial reactive oxygen species (ROS) production to explore the underlying mechanisms of periostin regulation in allergic inflammation.

## 2. Results

### 2.1. Quantitative Analysis of the Three Compounds Comprising GJ

**Figure 1:**
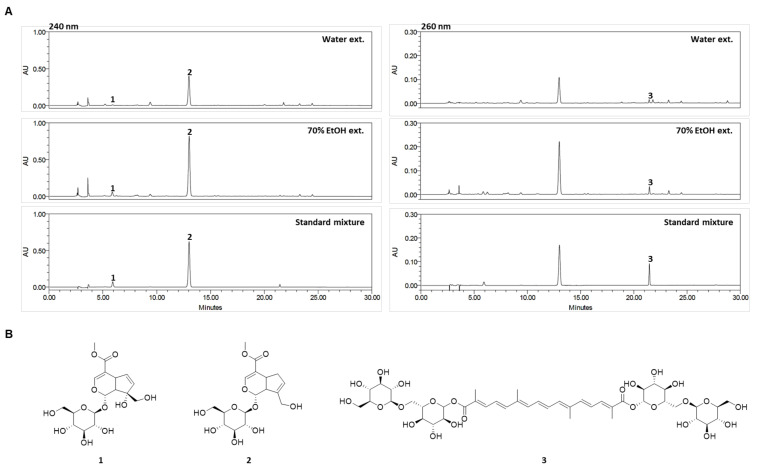
(**A**) High-performance liquid chromatography chromatograms of the water and 70% ethanol extracts of *Gardenia jasminoides* and standard mixture at 240 and 260 nm. (**B**) Chemical structures of the three standard compounds. 1: gardenoside, 2: geniposide, and 3: crocin.

GJ fruits extract has been reported to contain large quantities compounds, including gardenoside, geniposide, and crocin. HPLC was used for the simultaneous analysis of the GJ major active components gardenoside, geniposide, and crocin in water (GJW) and 70% ethanol (GJE) extracts. The three compounds were successfully separated within 22 min using mobile phases consisting of 0.1% (*v*/*v*) aqueous TFA and acetonitrile. The ultraviolet wavelengths used to detect the compounds were 240 nm for gardenoside and geniposide and 260 nm for crocin. The retention times of gardenoside, geniposide, and crocin were 5.87, 13.00, and 21.46 min, respectively. HPLC chromatograms for GJW, GJE, and the standard mixture are shown in Figure 1A, and the chemical structures of the three compounds are presented in Figure 1B. The calibration curves for the three standard compounds were calculated from the peak areas (*y*) of the standard solutions at various concentrations (*x*, μg/mL), ranging from 3.125 to 100 μg/mL for gardenoside and crocin and from 25 to 800 μg/mL for geniposide (Table 1).

The established HPLC method was applied for the quantitative analysis of gardenoside, geniposide, and crocin in GJ. The correlation coefficient values for the three compounds showed good linearity (*r*^2^ ≥ 0.9996). The ranges of the limits of detection and of quantification for the compounds were 0.124–0.347 μg/mL and 0.375–1.053 μg/mL, respectively. The amounts of the three standard compounds contained in GJW and GJE ranged from 3.59 to 129.64 mg/g and from 8.89 to 272.64 mg/g, respectively (Table 1). Among the three compounds, geniposide was the most abundant in both GJW and GJE (129.64 and 272.64 mg/g, respectively).

### 2.2. GJW and GJE Inhibit Allergy Symptoms and Reduce NALF Cell Number and Serum Markers in Ovalbumin-Induced AR Mice

**Figure 2:**
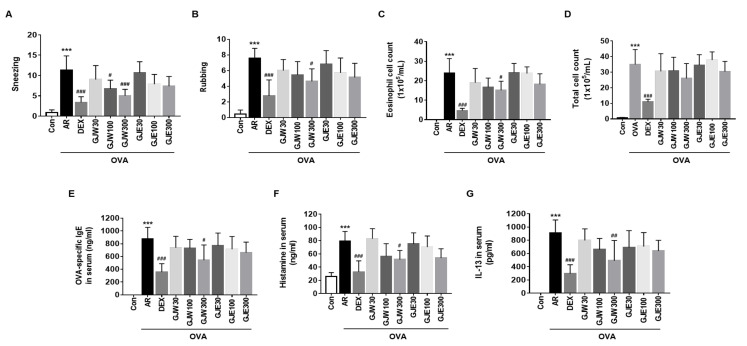
Effects of *Gardenia jasminoides* water (GJW) and 70% ethanol (GJE) extracts on allergy symptoms, nasal lavage fluid cells, and serum parameters. The AR mice were orally administered with GJW, GJE or DEX once daily, and anesthetized and euthanized 24 h after the last treatment. Allergy symptoms such as sneezing (**A**) and rubbing (**B**) scores were recorded at the time of the final intranasal treatment. Eosinophil (**C**) and total cell counts (**D**) were measured in the nasal lavage fluid. Serum levels of ovalbumin-specific immunoglobulin E (IgE) (**E**), histamine (**F**), and interleukin 13 (IL-13) (**G**) were evaluated using enzyme-linked immunosorbent assays. Data are presented as the means ± standard deviations (*n* = 6–8, each group). *** *p* < 0.001 vs. the non-sensitized (Con) group; ^#^ *p* < 0.05, ^##^ *p* < 0.01, and ^###^ *p* < 0.001 vs. the allergic rhinitis (AR) group. DEX, ovalbumin-sensitized with 1 mg/kg dexamethasone; OVA, ovalbumin.

To determine the anti-allergic effects of GJW and GJE in the ovalbumin-induced mouse model, we analyzed the behavior of the mice and changes in the number of NALF cells and serum levels of ovalbumin-specific IgE, histamine, and IL-13. As shown in Figure 2A,B, the AR group exhibited significantly more frequent sneezing and rubbing than the Con group, whereas these behaviors were significantly less frequent in the DEX group. Sneezing was significantly less frequent in both the GJW100 and GJW300 groups, whereas rubbing was less frequent only in the GJW300 group. The extract-administered groups tended to have lower eosinophilic and total cell numbers in the NALF than the other groups, with the number of eosinophils being significantly lower in the GJW300 group (Figure 2C,D). Moreover, serum levels of ovalbumin-specific IgE, histamine, and IL-13 tended to be lower in the extract-administered groups than in the AR group; the decrease was significant in the GJW300 group (Figure 2E–G). These data show that GJW and GJE ameliorated AR symptoms by reducing the expression of allergic inflammatory mediators such as IgE, histamine, and IL-13.

### 2.3. GJW and GJE Inhibit Periostin Levels and Eosinophil Infiltration in the Nasal Tissue of Ovalbumin-Induced AR Mice

**Figure 3:**
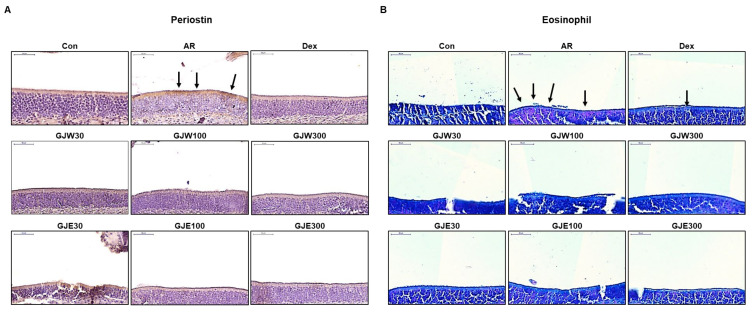
Effects of *Gardenia jasminoides* water (GJW) and 70% ethanol (GJE) extracts on histopathological changes in the nasal tissue. Immunohistochemical staining of periostin (**A**) and eosinophil staining (**B**) (upper left corner, scale bar = 50 μm). Con, non-sensitized; AR, allergic rhinitis; DEX, ovalbumin-sensitized with 1 mg/kg dexamethasone.

To evaluate the effects of GJW and GJE in the AR mouse model, we evaluated periostin levels and eosinophil expression in the nasal tissue. Periostin levels (brown stain [black arrow]) were elevated in the AR group compared to those in the NC group. However, in the groups administered GJW and GJE, periostin expression was lower than that in the AR group (Figure 3A). Histological staining of nasal tissue showed that eosinophil infiltration (red stain [black arrow]) was higher in the AR group than in the Con group, and that GJW and GJE decreased such infiltration (Figure 3B). These results suggest that GJW and GJE improved AR symptoms by modulating periostin and eosinophils, which are Th2 inflammatory mediators in the nasal mucosa.

### 2.4. GJW and GJE Inhibit the Activation of Differentiated Th2 Cells

**Figure 4:**
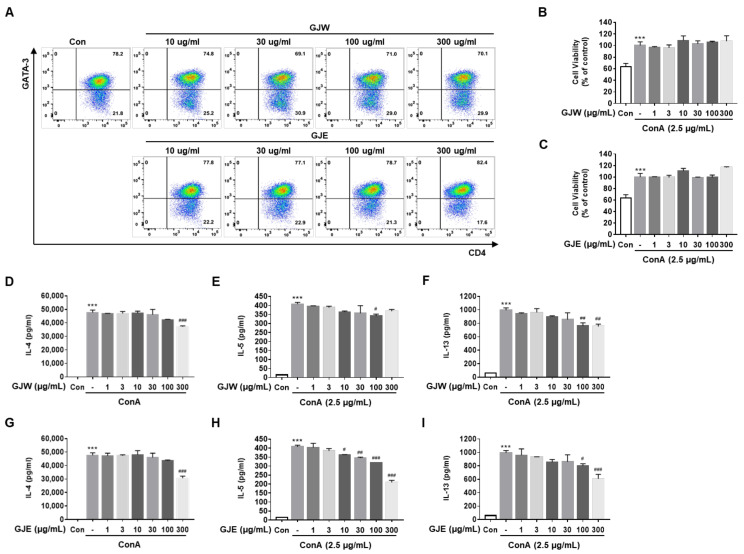
Effects of *Gardenia jasminoides* water (GJW) and 70% ethanol (GJE) extracts on the secretion of cytokines in differentiated Th2 cells. Naïve CD4+ T cells were differentiated into Th2 cells over 6 days. (**A**) The frequency of differentiated Th2 cells (CD3^+^ CD4^+^ GATA3^+^ cells) was examined using flow cytometry after treatment with GJW or GJE. (**B**,**C**) The viability of the differentiated Th2 cells was analyzed using EZ-Cytox solutions at the indicated concentrations of GJW or GJE. (**D**–**I**) The cytokine secretion levels in Th2 cells were assessed using LEGENDplex and flow cytometric analysis of the cell culture media. The data are presented as means ± standard deviations (*n* = 3). *** *p* < 0.001 vs. control; ^#^ *p* < 0.05, ^##^ *p* < 0.01, and ^###^ *p* < 0.001 vs. ConA. Con, control; ConA, concanavalin A; IL, interleukin.

First, we examined the changes in cell populations to determine whether GJW and GJE affect the differentiation of naïve CD4^+^ T cells to Th2 cells. The frequency of CD45^+^ CD3^+^ CD4^+^ CD44^-^ CD62L^+^ cells (naïve CD4^+^ T cells) was 14.4% in the spleen and lymph nodes, and the purity of naïve CD4^+^ T cells isolated from the spleen and lymph nodes was 85% (data not shown). The frequency of CD3^+^ CD4^+^ GATA3^+^ cells (i.e., differentiated Th2 cells) did not change following treatment with GJW and GJE compared to that in the control (Figure 4A). Next, we investigated the effects of GJW and GJE on the activation of differentiated Th2 cells; no cytotoxicity in differentiated Th2 cells due to GJW and GJE was observed at various concentrations of these extracts (Figure 4B,C). Differentiated Th2 cells secreted higher levels of concanavalin A-stimulated Th2 cytokines such as IL-4, IL-5, and IL-13 than control cells (Figure 4D–I). GJW and GJE significantly decreased the secretion levels of these cytokines at high concentrations (100 μg/mL and 300 μg/mL). These data show that GJW and GJE have an inhibitory effect on Th2 cells’ cytokine production but not on their differentiation and that these GJ extracts exhibit their anti-allergic effects by inhibiting major Th2 cytokines during allergy-associated inflammatory responses.

### 2.5. GJW and GJE Inhibit IL-4/IL-13-Induced Periostin Generation via the Regulation of Mitochondrial ROS Production in HNEpCs

**Figure 5:**
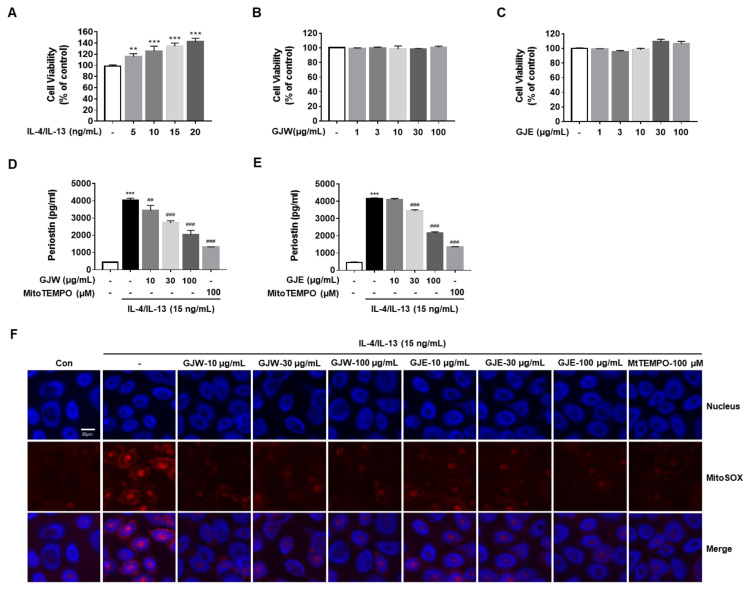
Effects of *Gardenia jasminoides* water (GJW) and 70% ethanol (GJE) extracts on interleukin (IL)-4/IL-13-induced periostin generation via mitochondrial reactive oxygen species (ROS) production in human nasal epithelial cells (HNEpCs). Cells were incubated in the presence of the indicated concentrations of IL-4/IL-13, GJW, or GJE for 24 h. (**A**–**C**) Cell viability was determined using the MTS assay. Cells were treated with GJW or GJE (10, 30, or 100 μg/mL) or MitoTEMPO (100 μM) along with 15 ng/mL IL-4/IL-13 conditioned medium for 24 h (**D**,**E**) or 1 h (**F**). (**D**,**E**) Periostin levels in the cell culture medium were measured using a competitive enzyme-linked immunosorbent assay following manufacturer’s instructions. (**F**) Analysis of mitochondrial ROS levels in IL-4/IL-13-treated HNEpCs. Cells were incubated with the mitochondrial indicator MitoSOX and analyzed using confocal microscopy (magnification: 130×, scalebar: 20 μm) after stimulation with 15 ng/mL IL-4/IL-13 in the presence or absence of GJW, GJE, or MitoTEMPO (the latter was used as a positive control for inhibiting the effects of IL-4/IL-13). Data are representative of three independent experiments and expressed as the means ± standard deviation; ** *p* < 0.01 and *** *p* < 0.001 vs. control; ^##^ *p* < 0.01; ^###^ *p* < 0.001 vs. IL-4/IL-13. MtTEMPO, MitoTEMPO.

We first determined the concentration-dependent cytotoxic effects of IL-4/IL-13, GJW, or GJE on HNEpCs. As shown in Figure 5A, IL-4/IL-13 induced the proliferation of HNEpCs in a dose-dependent manner. We then determined the non-toxic concentrations of GJW and GJE using our cell viability assay; as shown in Figure 5B,C, none of the concentrations of these extracts were cytotoxic. Next, we examined the effects of GJW and GJE on periostin production in IL-4/IL-13-treated HNEpCs. Similar to what was observed in the AR mouse model, elevated periostin levels were observed in HNEpCs; therefore, we performed our subsequent experiments using IL-4/IL-13 and analyzed periostin secretion into the cell culture medium. Under IL-4/IL-13-stimulated inflammatory conditions, GJW or GJE significantly suppressed periostin generation by HNEpCs in a dose-dependent manner; a similar inhibitory effect was observed when MitoTEMPO, a mitochondrial-targeted antioxidant and superoxide scavenger was used (Figure 5D,E). Our abovementioned experiments also showed that GJW and GJE inhibited IL-4/IL-13-induced periostin production in HNEpCs, mirroring the effects of MitoTEMPO. Therefore, to determine whether periostin synthesis, a key mediator in AR, is related to mitochondrial ROS, we assessed the effects of GJW and GJE on IL-4/IL-13-induced mitochondrial ROS production. As shown in Figure 5F, GJW and GJE decreased IL-4/IL-13-induced mitochondrial ROS production in a dose-dependent manner. These results indicate that mitochondrial ROS levels were markedly reduced by GJW or GJE after IL-4/IL-13 stimulation of HNEpCs. Taken together, these results show that GJW and GJE inhibit the production of periostin, an important mediator of inflammation, by decreasing mitochondrial ROS production in IL-4/IL-13-treated HNEpCs.

### 2.6. GJW and GJE Inhibit IL-4/IL-13-Induced Periostin Production via the Activation of UCP2 and p38 MAPK-ATF2 in HNEpCs

**Figure 6:**
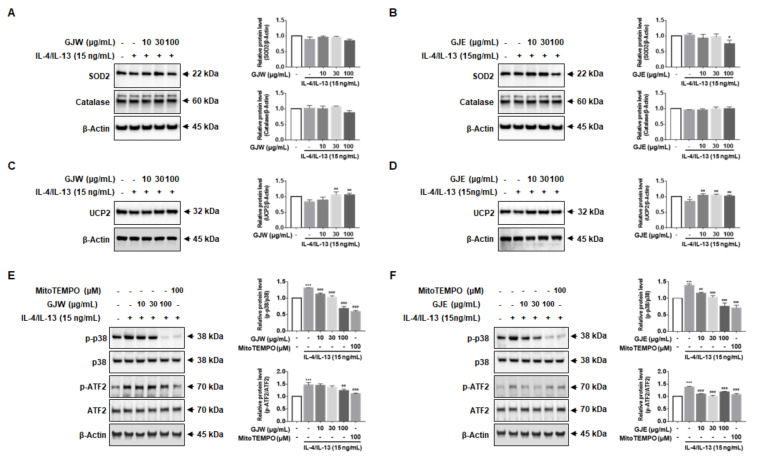
Effects of *Gardenia jasminoides* water (GJW) and 70% ethanol (GJE) extracts on the production of superoxide dismutase-2 (SOD2) and uncoupling protein-2 (UCP2), and on the activation of the p38-mitogen-activated protein kinase (MAPK)-activating transcription factor-2 (ATF2) signaling pathway in human nasal epithelial cells. (**A**–**F**) Cells were treated with GJW and GJE (10, 30, and 100 μg/mL) or with MitoTEMPO (100 μM) along with 15 ng/mL IL-4/IL-13 for 1 h. Total cell lysates were analyzed via western blotting using specific antibodies for SOD2 (**A**,**B**), catalase (**A**,**B**), UCP2 (**C**,**D**), phospho-p38, p38, phospho-ATF2, and ATF2 (**E**,**F**); total β-actin served as a loading control. Images were quantified using ImageJ. Data are representative of three independent experiments and expressed as the means ± standard deviations; * *p* < 0.05 and *** *p* < 0.001 vs. control; ^#^ *p* < 0.05, ^##^ *p* < 0.01, and ^###^ *p* < 0.001 vs. IL-4/IL-13. p-p38, phospho-p38; p-ATF2, phospho-ATF2.

Given the effects of GJW and GJE on mitochondrial ROS production, we investigated whether GJW and GJE-mediated inhibition of ROS production is regulated by antioxidant enzymes. We found that GJW and GJE did not increase the expression levels of SOD2 or catalase (Figure 6A,B), suggesting that the effects of GJ extracts on mitochondrial ROS were not dependent on SOD2. Therefore, we next measured the expression of UCP2. As shown in Figure 6C,D, IL-4/IL-13 reduced UCP2 protein levels, whereas treatment with GJW and GJE prevented this cytokine-induced decrease in UCP2 expression. These results show that the inhibitory function of GJW and GJE against IL-4/IL-13-induced mitochondrial ROS production is mediated through UCP2. Next, we investigated whether GJW and GJE mediate mitochondrial ROS and MAPK activation. As shown in Figure 6E,F, IL-4/IL-13 increased the phosphorylation of p38-MAPK and enhanced the expression of ATF2 (which is downstream of p38-MAPK) in HNEpCs, whereas treatment with GJW and GJE inhibited the phosphorylation of p38 and ATF2 in a concentration-dependent manner. Taken together, these results suggest that inhibition of IL-4/IL-13-induced periostin production by GJW and GJE in HNEpCs is at least partially mediated by mitochondrial ROS-, p38-MAPK-, and ATF2-dependent mechanisms.

## 3. Discussion

In this study, we investigated the effects of extracts from GJ on AR-associated inflammatory responses that cause epithelial damage. Our results show that GJW and GJE are effective inhibitors of AR and that they function by significantly reducing inflammatory cell infiltration, Th2 cytokine production, eosinophil migration, mitochondrial ROS production, periostin production, and p38-MAPK/ATF2 activation. AR is a hypersensitive inflammatory condition in the nasal mucosa that occurs following the activation of nasal epithelium Th2 immune cells and eosinophils by specific allergens, resulting in persistent inflammation. Therefore, nasal epithelium-mediated immune response is an important target for AR-treating therapeutics [4,5].

Previous studies have shown that GJ extracts have anti-inflammatory and antioxidant properties [26,27,33,34], and are effective against allergic inflammatory diseases such as asthma and atopic dermatitis [30,31]. Based on these findings, we investigated allergic inflammatory mediators, immune cells including eosinophils, and their underlying mechanisms to understand how GJW and GJE inhibit AR inflammation-like conditions in an ovalbumin-induced AR mouse model and in IL-4/IL-13-treated (i.e., inflammatory pathway-activated) HNEpCs. Our results showed that GJW and GJE inhibit Th2 immune cells and periostin, the latter being a key biomarker in allergic inflammatory responses, by attenuating mitochondrial ROS/p38-MAPK/ATF2 mediated signaling. As such, these extracts appear to be effective against AR and potentially other upper airway inflammatory disorders.

AR increases the levels of Th2 cytokines, IgE, histamine, chemokine (C-C motif) ligand 26/eotaxin-3, and periostin, which are secreted and recruited by immune and epithelial cells in the nasal mucosa [6,7,35]. Among them, periostin (osteoblast-specific factor 2), like eosinophils, is an important regulator of the Th2 inflammatory response in individuals with allergic diseases. This protein is mainly secreted by fibroblasts and epithelial cells stimulated by Th2-associated inflammatory cytokines such as IL-4 and IL-13 [19,20,24]. Moreover, periostin usually modulates fibrosis and airway tissue remodeling in allergic inflammatory responses, and increases superoxide anion generation, transforming growth factor-β generation, and eosinophil infiltration; it also elevates IgE-dependent degranulation responses in mast cells [20,36,37]. Th2 cytokines, IgE-histamine, periostin, and eosinophils contribute to regulating the AR response; we found that GJW and GJE counteracted these inflammatory mediators and reduced behaviors associated with AR (such as sneezing and rubbing) in our ovalbumin-induced AR mouse model (Figure 2, Figure 3 and Figure 4).

ROS is an important second messenger in inflammatory responses; moreover, the mitochondria is a major source of intracellular ROS. The disproportionate production of ROS, which is an important signaling molecule in both metabolic and immune system processes, causes inflammation and oxidative tissue damage. In particular, mitochondrial ROS plays a prominent role in inducing inflammatory mediators and immune regulation events in various inflammation-related diseases [38,39,40]. Mitochondrial ROS affects inflammatory cytokine production via the activation of MAPK, including extracellular signal-regulated kinase, c-Jun n-terminal kinase, and p38 [41]. Based on this evidence, we investigated the mechanisms underlying the activities of these extracts in HNEpCs and found that GJW and GJE significantly inhibited the upregulation of mitochondrial ROS and periostin in IL-4/IL-13-treated HNEpCs in a dose-dependent manner, mirroring the effect of MitoTEMPO because these extracts function as mitochondria-targeted antioxidants (Figure 5). Moreover, we found that GJW and GJE inhibit antioxidant enzymes such as SOD2 and catalase. Notably, the level of SOD protein decreased only slightly when treating the cells with 100 μg/mL of GJW and/or GJE; these data suggest that GJW and GJE do not require SOD to reduce the superoxide anion.

We also showed that GJW and GJE increase the expression of UCP2, which is a component of another mitochondrial ROS-associated signaling pathway [42,43,44]. Located in the inner mitochondrial membrane, UCP2 functions to protect against oxidative stress by regulating the production of superoxide anions [42,44,45,46]. Our findings illustrated that GJW and GJE reduce mitochondrial ROS via the upregulation of UCP2, not by regulating antioxidant enzymes per se. Moreover, GJW and GJE treatment reduced periostin secretion by inhibiting p38-MAPK activation and ATF2, the latter being a member of the ATF/CREB transcription factor family that acts downstream of p38-MAPK and mediates IL-4/IL-13-induced inflammatory responses in HNEpCs. Ultimately, our study demonstrated that GJW and GJE act as effective inhibitors of the allergic inflammatory response in IL-4/IL-13-stimulated HNEpCs; that is, these extracts inhibit the mitochondrial ROS-mediated induction of periostin by increasing UCP2 expression, which in turn attenuates p38-MAPK/ATF2 signaling (Figure 6). The GJ extracts used in our study consisted of at least three active compounds; therefore, further investigations are warranted to elucidate the specific mechanisms underlying the activity of GJ extracts and their major components (such as gardenoside, geniposide, and crocin) and determine how the mitochondrial ROS/p38-MAPK/ATF2 pathway is involved. In our future studies, we will investigate the side effects possibly associated with GJ extract and its main components to facilitate future clinical trials.

## 4. Materials and Methods

### 4.1. Preparation of GJ Extracts

GJ fruits, their producing district being Wando-gun, Jeollanam-do, Republic of Korea, were purchased from Mega Herb Co. (Chungbuk, Korea), and a voucher specimen was deposited at the Herbal Medicine Research Division of the Korea Institute of Oriental Medicine (Daejeon, Korea). The dried GJ (100 g) was extracted with 70% ethanol (1 L) and water (1 L) at 25 °C for 2 h, respectively. The extracted solutions were sequentially filtered through 5-μm and 1-μm filters and then dried under reduced pressure to obtain the powdered 70% ethanol extract (16.92 g corresponding to 16.92%) and water extract (34.25 g corresponding to 34.25%).

### 4.2. High-Performance Liquid Chromatography (HPLC) Analysis

#### 4.2.1. Chemicals and Reagents

Three standard compounds (purity ≥ 98.0%) of GJ gardenoside, geniposide, and crocin (ChemFaces Biochemical Co., Ltd., Wuhan, China) were used for HPLC analysis. HPLC grade acetonitrile, methanol, and water were purchased from J. T. Baker Chemical Co. (Phillipsburg, NJ, USA), and trifluoroacetic acid (TFA) was obtained from Sigma-Aldrich (St. Louis, MO, USA).

#### 4.2.2. Preparation of Sample and Standard Solutions

GJ water (GJW) and 70% ethanol (GJE) extracts were dissolved in methanol to a concentration of 2 mg/mL and passed through a syringe filter (0.45 μm pore size) for quantitative analysis. Three standard compounds were dissolved in methanol at 1 mg/mL; these stock solutions were mixed and diluted with methanol to obtain a series of standard mixtures for quantitative analysis.

#### 4.2.3. Apparatus and Chromatographic Conditions

To perform quantitative analysis of the three GJ compounds, a Waters Alliance e2695 system (Waters Corp., Milford, MA, USA) equipped with a photodiode array detector (#2998; Waters Corp.) was used; the ultraviolet wavelength ranged from 190 to 400 nm. The data were collected and processed using the Empower software (version 3; Waters Corp.). The chromatographic separation of the three compounds was conducted on a Sunfire C_18_ analytical reversed-phase column (250 × 4.6 mm, 5 μm, Waters Corp.) that was maintained at 30 °C. The mobile phases consisted of 0.1% (*v*/*v*) aqueous TFA (A) and acetonitrile (B), and the gradient elution was as follows: 10–15% B for 10 min, 15–45% B for 20 min, 45–100% B for 10 min, 100% B for 10 min, and 10% B for 10 min. The flow rate of the mobile phase and injection volume of the sample were 1 mL/min and 10 μL, respectively.

### 4.3. Animals

Specific pathogen-free BALB/c female mice (6 weeks old, weighing 18–20 g) were obtained from Samtako Bio Korea (Kyung Gi-Do, Korea). The animals were maintained in an animal room with controlled conditions (temperature, 22 °C ± 2 °C; humidity, 55% ± 15%; and light/dark cycle, 12/12 h). The experimental protocols were approved by the Institutional Animal Care and USE committee of Chonnam National University Laboratory Animal Center (approval number CNU IACUC-YB-2020-77, 7 September 2020).

### 4.4. AR Mouse Model and Treatments

After an adaptation period of 1 week, the mice were sensitized on days 0, 7, and 14 by intraperitoneal injections with 50 μg ovalbumin (Sigma-Aldrich) and 2 mg aluminum hydroxide (Sigma-Aldrich) dissolved in 200 μL of phosphate-buffered saline (PBS). On day 21, the mice were randomly divided into nine groups (*n* = 6–8 per group), including the Con group (non-sensitized), AR group (ovalbumin-sensitized), DEX group (ovalbumin-sensitized with 1 mg/kg dexamethasone), GJW group (ovalbumin-sensitized with GJW 30, 100, and 300 mg/kg/mice), and GJE group (ovalbumin-sensitized with GJE 30, 100, and 300 mg/kg/mice). The treatment groups were orally administered GJW, GJE or DEX once daily on day 21 to day 27. The mice were challenged on days 21, 23, 25, and 27 via intranasal administration of 400 μg ovalbumin; they were anesthetized and euthanized on day 28 (after 24 h of last oral administration) using Alfaxan (Jurox Pty Ltd., Rutherford, Australia).

### 4.5. Allergic Symptoms of AR Mouse Model

One hour after the last intranasal treatment, the number of times the mice sneezed and rubbed their noses were counted for 5 min.

### 4.6. Analysis of Serum and Nasal Lavage Fluid (NALF)

Blood was collected from the abdominal vein of anesthetized mice using Alfaxan (Jurox Pty Ltd.), and the levels of ovalbumin-specific IgE (500840, Cayman Chemical, Ann Arbor, MI, USA), histamine (ENZ-KIT140-0001, Enzo Life Science, Farmingdale, NY, USA), and IL-13 (DY413, R&D Systems, Abingdon, UK) in serum were measured using commercial enzyme-linked immunosorbent assay (ELISA) kits following manufacturers’ instructions. NALF was collected by washing the nasal cavity with 10 mL of PBS after creating an incision in the bronchial tubes. The total number of cells in the NALF was measured using a cell counter (Thermo Fisher Scientific, Waltham, MA, USA). After centrifugation using Cytospin (Hanil, Seoul, Korea), Diff-Quik staining (Sysmex, Chuo-ku, Kobe, Japan) was performed according to the manufacturer’s instructions, and the numbers of eosinophils in each group were measured and compared using a light microscope (Olympus, Tokyo, Japan).

### 4.7. Immunohistochemistry

The excised heads of mice were fixed in 10% formalin solution (Sigma-Aldrich) for 1 week and then placed in 0.1 M EDTA buffer (Biosolution Co. Ltd., Seoul, Korea) for 2 weeks to demineralize the skull tissue. After paraffin embedding, nasal tissues were cut into 4-μm thick sections using a microtome. The nasal tissue sections were then deparaffinized and rehydrated, after which immunohistochemical staining was performed using periostin polyclonal antibody (PA5-34641, Thermo Fisher Scientific). The nasal tissue section was incubated with periostin polyclonal antibody diluted 1:100 in antibody diluent (S0809, Dako, Agilent Technologies, Inc., Santa Clara, CA, USA) overnight at 4 °C; after subsequent washing, the tissue was incubated with biotinylated secondary antibody (MP-7801-15, Vector Labs, Burlingame, CA, USA) at room temperature for 30 min. The sections were scanned with slide digital scanners Pannoramic DESK (3DHISTECH, Budapest, Hungary), and periostin level changes were observed using the Pannoramic Viewer (3DHISTECH).

### 4.8. Histological Analysis

Histological analysis was performed by staining the eosinophils using the combined eosinophil-mast cell kit (ab150665, Abcam, Cambridge, UK) per the manufacturer’s instructions. The sections were scanned with slide digital scanners Pannoramic DESK (3DHISTECH), and eosinophil infiltration changes were observed using the Pannoramic Viewer (3DHISTECH).

### 4.9. Isolation of Naïve CD4^+^ T Cells and Differentiation of Th2 Cells

Five-week-old male BALB/c mice (weighing 18–20 g) were obtained from Japan SLC, Inc. (Shizuoka, Japan) and maintained in a controlled, specific pathogen-free environment (temperature, 24 °C ± 2 °C; humidity, 50% ± 5%; and light/dark cycle, 12/12 h with food and water provided *ad libitum*). Spleen tissues were passed through 40-μm cell strainers (Falcon, New York, NY, USA), and red blood cells were removed using a specific lysis buffer (BioLegend, San Diego, CA, USA). Naïve CD4^+^ T cells were isolated from the splenocytes using the Naïve CD4^+^ T Cell Isolation Kit (MACS; Miltenyi Biotec Inc., Auburn, CA, USA) and were differentiated into Th2 cells using the CellXVivo Mouse Th2 Cell Differentiation Kit (R&D Systems, Minneapolis, MN, USA) according to the manufacturers’ instructions. The animal experiments for the differentiation of Th2 cells from naïve CD4+ T cells were approved by the institutional animal care committee of Korea Institute of Oriental Medicine (KIOM: #20-070).

### 4.10. Evaluation of Th2 Cell Differentiation

The naïve CD4+ T cells were treated with various concentrations of GJW or GJE during the differentiation of Th2 cells. The differentiated Th2 cells were then stained with the following antibodies obtained from BD Bioscience (Franklin Lakes, NJ, USA): anti-CD3-FITC (Cat. No. 561798), anti-CD4-BB700 (Cat. No. 566407), and anti-GATA3-BV421 (Cat. No. 563349). The differentiated Th2 cells were quantified using an LSRFortessa^TM^ X-20 flow cytometer (BD Biosciences) and analyzed using FlowJo software version 10 (FlowJo, Ashland, OR, USA).

### 4.11. Analysis of Th2 Cell Activation and Viability

The differentiated Th2 cells (5 × 10^6^ cells/mL) were treated with various concentrations of GJW or GJE and stimulated with 2.5 μg/mL concanavalin A (Sigma-Aldrich). After 24 h, the culture media were analyzed for secreted Th2 cytokines (IL-4, IL-5, and IL-13) using the LEGENDplex Mouse Th Cytokine Panel (BioLegend, Cat. No. 741044), and cell viability was determined using the EZ-Cytox solution (DoGenBio Co. Ltd., Seoul, Korea) according to the manufacturers’ instructions.

### 4.12. Cell Culture

HNEpCs purchased from PromoCell GmbH (Heidelberg, Germany) were cultured under standard conditions in airway epithelial cell growth medium (PromoCell) at 37 °C in a humidified 5% CO_2_ incubator. For all experiments, cells of early passage numbers (six or less) were used.

### 4.13. Determining Cell Viability

HNEpCs were seeded into 96-well plates at a density of 1 × 10^4^ cells/well and incubated in airway epithelial cell basal (AECB) medium (PromoCell) prior to treatment with different concentrations of recombinant human IL-4/IL-13 (Peprotech, Rocky Hill, NJ, USA) or GJW/GJE. After 24 h of incubation, cell viability was evaluated using the MTS assay (Promega, Madison, WI, USA), according to manufacturer’s instructions. In brief, the culture medium in each well was replaced with 100 μL of medium containing MTS solution and incubated for 4 h at 37 °C. The absorbance of the wells was measured with a microtiter plate reader (BIO-TEK, Synergy HT, Winooski, VT, USA) at 490 nm. Cell viability was calculated using the optical density of each treatment group and expressed as a percentage of the control cell optical density.

### 4.14. Determination of Protein Levels

HNEpCs were seeded at 2.5 × 10^5^ cells/well in 6-well plates 24 h before treatment with 15 ng/mL IL-4/IL-13 (7.5 ng/mL of each) in the presence or absence of GJW, GJE, or Mito-TEMPO (Sigma-Aldrich) for 1 h. The HNEpCs were then lysed with Laemmli sample buffer (Bio-Rad, Hercules, CA, USA) and heated at 100 °C for 5 min. Next, 25–30 µg of these extracted proteins was subjected to sodium dodecyl sulfate-polyacrylamide gel electrophoresis under denaturing conditions. The separated proteins were then transferred to nitrocellulose membranes (Bio-Rad) using a tank blotting apparatus (Bio-Rad). The protein-blotted membranes were probed with specific primary antibodies against superoxide dismutase (SOD), catalase, uncoupling protein-2 (UCP2), phospho-p38, p-38, phospho-activating transcription factor-2 (ATF2), and β-actin (Cell Signaling Technology, Danvers, MA, USA; 1:1000 dilution), washed with tris-buffered saline containing 0.1% Tween-20, and incubated with horseradish peroxidase-linked secondary antibodies. After the membranes were washed three times, immunoreactivity was detected using the EzWestLumiOne enhanced chemiluminescence solution (Atto Corporation, Tokyo, Japan); protein bands were then visualized with ChemiDoc (Bio-Rad). Quantification of western blot images were performed using ImageJ 1.52a software (National Institutes of Health, Bethesda, MD, USA).

### 4.15. Immunofluorescence and Confocal Microscopy

HNEpCs were cultured on glass-bottom dishes (Thermo Fisher Scientific) and treated with various concentrations of GJW and/or GJE or with 100 μM of MitoTEMPO for 1 h after stimulation with 15 ng/mL IL-4/IL-13. The cells were then washed twice with PBS and incubated with AECB medium containing 5 μM MitoSOX (Thermo Fisher Scientific) at 37 °C for 20 min. After incubation, cells were washed again with Hank’s Balanced Salt Solution, and the cell nuclei were stained with DRAQ5 (Cell Signaling) in PBS. After a final incubation of 5 min, the cells were analyzed under an FV10i confocal microscope (Olympus).

### 4.16. Measurement of Periostin

Periostin concentrations were measured using a competitive ELISA kit (R&D Systems, Minneapolis, MN, USA) following the manufacturer’s protocol. HNEpCs were seeded in 96-well plates at a density of 1 × 10^4^ cells/well and incubated in AECB medium before exposure to IL-4/IL-13 (15 ng/mL) in the presence or absence of GJW, GJE, or MitoTEMPO for 24 h. Periostin secreted into the media was measured in triplicate against standards reconstituted in medium using the ELISA kit. The results were normalized to the controls.

### 4.17. Statistical Analysis

Data are presented as means ± standard deviations, and statistical analyses were performed using GraphPad prism software version 7 (GraphPad Software, Inc., La Jolla, CA, USA). Significant differences between groups were determined using one-way analysis of variance, followed by Tukey’s multiple comparison test. Differences were considered statistically significant at *p* < 0.05.

## 5. Conclusions

We report, to the best of our knowledge, for the first time that GJ extracts function as effective inhibitors of AR, including allergy-associated secretion of Th2 cytokines, immune cell activation, and inflammatory mediator upregulation. The beneficial therapeutic effects of GJ extracts on AR were elicited via the inhibition of the p38-MAPK/ATF2 signaling pathway. Treatment with GJ extracts suppressed IgE, histamine, IL-13, eosinophil, and periostin levels in ovalbumin-induced AR mice. Additionally, GJ extracts inhibited IL-4/IL-13-induced mitochondrial ROS-induced periostin production and reduced p38-MAPK/ATF2 activity in HNEpCs. These results suggest that GJ extracts could ameliorate the symptoms of AR caused by excess periostin and immune cell production, illustrating the potential therapeutic properties of GJ extracts against allergic diseases as well as AR.

## Figures and Tables

**Table 1:** Regression equation, linearity, LOD, LOQ, and contents of the three compounds.

Compound	Linear Range (μg/mL)	Regression Equation (*y* = a*x* + b) ^(a)^		*r* ^2^	LOD ^(b)^ (μg/mL)	LOQ ^(c)^ (μg/mL)	Content (mg/g)	
		Slope (a)	Intercept (b)				70% EtOH	Water
Gardenoside	3.125–100	12175	7278.8	0.9997	0.313	0.950	21.24 ± 0.08	3.59 ± 0.02
Geniposide	25–800	15204	93731	0.9996	0.347	1.053	272.64 ± 0.21	129.64 ± 0.49
Crocin	3.125–100	8965.4	3561.9	0.9999	0.124	0.375	8.89 ± 0.00	3.84 ± 0.03

**^(a)^***y* = a*x* + b, *y* means peak area and *x* means concentration (μg/mL); **^(b)^** LOD (limit of detection): 3.3 × (standard deviation of the response/slope of the calibration curve); **^(c)^** LOQ (limit of quantification): 10 × (standard deviation of the response/slope of the calibration curve).

## Data Availability

Data is contained within the article.
